# Co-Operative Production and Profit-Sharing

**Published:** 1891-08-08

**Authors:** 


### Co-operative Production and Profit-Sharing.

We have heard a good deal of late, and we are destined to hear a good deal more, of schemes for rescuing such of our fellow-creatures as, through their own fault, or by the cruel pressure of circumstances, have sunk so low that they seem to have lost both chance and power to recover their footing. Such schemes deserve, no doubt, the earnest consideration of the English public. Meanwhile, it would be very unfortunate if, in our anxiety to stretch out hands of help to the "submerged tenth," we lost sight of another duty—that of giving encouragement and aid to those among our working brethren who are manfully trying to raise themselves.

The Labour Association for Promoting Co-operative Production* has for the last four years been doing excellent work in a quiet and unostentatious way by recommending to employers and employés alike a method of reconciling the interests of capital and labour, and securing for the latter, without injury to the former, a direct interest in the profits of the business, which we cannot doubt is the most hopeful that has yet been devised. As the report of the Association says, it aims "not so much to raise our working people as to help them to raise themselves, thinking this more effectual than charity, and more practical than any form of State Socialism." It does not subsidise co-operative societies, but helps them at the outset to organise their working arrangements, gives lectures, both to societies already formed and to the general public, and takes every opportunity of enforcing the teaching of practical experience as to the benefits to be gained by this new method of business. Some of our readers may, perhaps, have attended the Co-operative Festival held at the Crystal Palace last August, when there was an exhibition of various products of co-operative industry—cutlery, hosiery, cocoa, boots and shoes, woollen cloth, and many other things, the exhibition being organised by this same Labour Association.

There are two kinds of Co-operation—indeed, there are three, if we must count that species with which such enterprises as the Civil Service and the Army and Navy Stores have made us familiar. These latter, however, are merely concerned with co-operation between dealer and consumer. The sort of co-operation which the Labour Association seeks to establish is, as its name implies, *labour* co-operation—that is, a participation in the profits of a business by all those who labour in it; and of this sort of co-operation there are, as has been said, two kinds—profit-sharing and co-operative production.

The term profit-sharing speaks for itself. At the end of the year, the gross receipts of the business are portioned out, so much for raw material, rent, wages, salaries, interest on capital, &c., and, finally, so much for the employés, to be divided among them in proportion to their normal wage. All kinds of different systems prevail in the various profit-sharing businesses. In some, the workman's bonus is paid in cash; in some, it goes to form a provident fund; in others, part goes to the provident fund, part is paid out in cash. Again, while some firms only give their employés an indefinite share of the profits, which is fixed upon at the end of each year, others give a definite percentage, there being, once more, no kind of uniformity as to the scale of this percentage. Some firms only offer two or three per cent., others, ten, twenty-five, thirty, and even fifty per cent.—though it may be noted that two at least of those offering the latter percentage have never had any profits to divide. It has often been remarked that profit-sharing can never be a thoroughly satisfactory method of doing business—that it is, in fact, unfair to the master, for, while the men participate in the profits, they cannot, in the nature of things, participate in the loss which a bad year may bring. It seems, in fact, rather too much of a "Heads I win, tails you lose" arrangement to suit the tastes of some employers. The Labour Association makes an ingenious attempt to get over the difficulty by calling the system profit *and loss* sharing, and pointing out that in bad years the workers may obtain no reward for the extra zeal and intelligence which they have thrown into their work. This consideration is, indeed, not without force; and although we can hardly admit that the workers' chance of loss is really proportionate to the master's, still, the undoubted fact that profit-sharing does stimulate the industry of the workers, and thus increase the chance of profit, makes the system well worth a trial.

Co-operative production, on the other hand, gives the employé his fair share of risk, he being encouraged to take shares in the business, and thus become, in fact, a capitalist as well as a labourer. This, we think, is the most satisfactory kind of co-operation where practicable, for when labour and capital are thus combined it is obvious that there can hardly be a strife between them. Many co-operative societies combine co-operative production and profit-sharing, as, for instance, the Welsh Slate Company (where eighty out of the two hundred men employed are shareholders) and the Kettering Boot and Shoe Society, which, in 1889, paid eighteenpence in the pound on wages, and ten per cent. on share capital, besides fourpence in the pound to those who purchased goods from it.

Now, what are the advantages which co-operation may be expected to bring in its train? First of all, it is claimed for it—and claimed, we are sure, with truth—that it greatly stimulates the energy of the workers, and is, therefore, highly successful, merely from an economical point of view. Secondly, it tends to prevent friction between employers and employed. True, it will probably be in the memory of all our readers that the Metropolitan Gasworkers' Strike originated in a dispute over a proposed profit-sharing agreement, and this at first sight would seem to militate against the truth of our statement. But the fact of the matter is this: profit-sharing should not be used by the masters as a means of fettering the liberty of action of his employés, whether in the way of preventing them from leaving his service, through the dread of losing their accumulating bonus, or in the way of formal agreements that there shall be no strikes. Try to make it serve this purpose, and it will create distrust rather than allay it. But use it in its natural way, as a means of giving the workman a direct interest in the success of the business, and it becomes, as has been said by a firm which has tried it, "a lubricant which can scarcely be over-estimated."

Lastly, co-operation is most valuable as allaying the dissatisfaction with the circumstances of their daily life and labour which is so prevalent among the working classes now-a-days. It may be an "idea" that mere wage-earning is inconsistent with the true dignity of labour; but, after all, we live by our ideas, and are happy in proportion as we can shape our lives in accordance with them. And if industrial partnership appears—as without doubt it does appear—infinitely more attractive to the working-man than any system of wage-earning, why not help him in his endeavours to secure it? It is, indeed, greatly for our interest, as well as for his own, that these endeavours should be successful. We can all do something to help by giving our patronage to productive societies (which, as the Association frankly declares, "want custom"), if we can do nothing else; and those who have a little money to spare might do far worse with it than become subscribers to the association—or take shares in a co-operative society, thus "combining philanthropy with profit," as we English people—or else we are slandered—dearly love to do.

* Central Office, 1, Norfolk Street, Strand. Secretary, Mr. H. E. Ivimey.

